# Could Insect Products Provide a Safe and Sustainable Feed Alternative for the Poultry Industry? A Comprehensive Review

**DOI:** 10.3390/ani13091534

**Published:** 2023-05-03

**Authors:** Ayman Khalifah, Sara Abdalla, Mai Rageb, Lucianna Maruccio, Francesca Ciani, Karim El-Sabrout

**Affiliations:** 1 Livestock Research Department, Arid Lands Cultivation Research Institute, City of Scientific Research and Technological Applications (SRTA-City), New Borg El-Arab 21934, Egypt; 2 Plant Protection and Biomolecular Diagnosis Department, Arid Lands Cultivation Research Institute, City of Scientific Research and Technological Applications (SRTA-City), New Borg El-Arab 21934, Egypt; 3 Department of Food Technology, Arid Lands Cultivation Research Institute, City of Scientific Research and Technological Applications (SRTA-City), New Borg El-Arab 21934, Egypt; 4 Department of Veterinary Medicine and Animal Production, University of Naples Federico II, 80138 Naples, Italy; 5 Department of Poultry Production, Faculty of Agriculture, Alexandria University, Alexandria 21545, Egypt

**Keywords:** edible insect products, egg production, growth, gut health, immunity, meat quality, public health, welfare

## Abstract

**Simple Summary:**

Insects are among the most numerous and abundant animal species on the planet. They are currently regarded as the most promising and sustainable source of animal protein, owing to their abundance and nutritional value. Edible insect products are considered eco-friendly nutraceuticals that have several positive effects on poultry production, such as improved body weight for broilers and egg production in layers. Therefore, these products have the potential to be sustainable food ingredients for poultry farming, based on the information presented in this review.

**Abstract:**

The planet is home to more than 2000 species of edible insects, some of which have been consumed as food for many years. Recently, edible insect products have been gradually increasing in several countries, such as Italy and Egypt, as novel feed resources for humans and animals due to their availability, potential economic benefits, and high nutritive value. The insect industry can provide a new solution for livestock nutrition and offer many additional advantages, but there are obstacles to overcome, such as some nutritional organizations that forbid its usage. Nevertheless, previous research indicates that different insect species could be used safely as nutraceuticals in poultry farming to improve broiler growth performance (>3%) and layer egg production (>5%). Among these species, there are various products and extracts that can be used in poultry nutrition in a sustainable manner. This review provides an outline of insect composition, nutrient values, application in poultry feed, safety, and guidelines, and finally, the future perspectives of insects as an alternative feed source in poultry diets.

## 1. Introduction

Poultry production is the most important sector of animal manufacturers and the agricultural sub-sector with the fastest growth, particularly in developing countries [1]. Finding alternatives to traditional livestock and feed sources is critical to address the growing population issue in some countries [2,3]. On the other hand, seeking alternative protein sources for poultry farming can help to reduce the ecological footprint and improve animal welfare. Thus, to overcome this obstacle, the poultry industry needs to concentrate more on sustainable production practices and alternative sources of protein [4].

The expenses of feed are estimated to be between 65 and 75% of the total cost of producing poultry [5]. The traditional source of protein in poultry feed is soybean meal [4]. However, since 2005, there has been a lack of plant-based protein sources, particularly soybean meal [6]. This lack is due to the absence of resources being created to meet the rising demand, which is causing a reduction in the amount of available land, deforestation, and the present trend of using soybean oil for the manufacturing of biofuels [7]. Because the cost of common feedstock is steadily rising, this has led to pressure not only on the ecosystem but also on the economy. There have been numerous initiatives to keep the cost of feeding to a bare minimum. To support the sustainable development of poultry production, these initiatives included substituting the pricey feedstock with more affordable, effective, and plentiful alternative sources [8,9].

**Figure 1:**
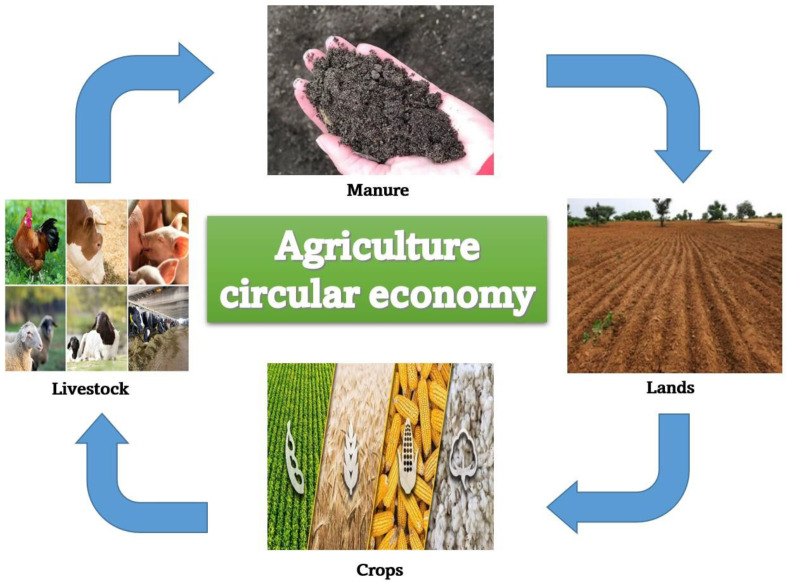
Example of a circular agriculture economic plan.

In Europe, a new agricultural circular economic action plan was proposed by the European Commission in March 2020 as part of the European Green Deal as a necessity for achieving the European Union (EU) 2050 climate independence objective and environmental sustainability [10]. This strategy contains legislative initiatives to deal with waste, with the long-term objectives of reducing the generation of waste and promoting reuse and recycling. In order to get rid of loops in the lifecycles of products, the strategy also supports the circular economy in agriculture at every stage of the value chain, from production to spending, maintenance and production, waste management, and feedback of secondary raw materials. This concept may differ from one continent to another depending on the needs of the poultry sector and the availability of raw materials. However, the European sustainable production management principle is “grow, make, use, and restore”, or the flow of material through the system that requires minimal external inputs, recycling of resources, and producing as little waste, emissions, or pollution as possible. This is compared to the linear economic principle of manufacturing, which is based on “take, make, use, and dispose of” [11]. All parts of the circular chain must be optimized, linked, and matched to each other for a circular agricultural system to be economically successful. An example of a circular agriculture economic strategy is shown in Figure 1.

Insects are among the most numerous and abundant animal species on the planet. They can deal with the aforementioned issues, making them a desirable and vital participant in a sustainable future. Insects can be grown in vertical farming systems and they require less space than the current sources of protein because they frequently require less water, which reduces their water usage [12,13]. Around 9.5% of the world’s agricultural output is pollinated by insects, which also produce 15–35% of the feed for livestock and humans and around 40% of the world’s nutritional supply for individuals [14,15]. Insects can act as the “missing link” to close product lifecycle loops, ensuring the long-term sustainability of the food chain [16]. The pertinent insect species can be grown on organic side streams and have a high conversion efficiency [17]; they release fewer greenhouse gases and ammonia than traditional livestock depending on the rearing substrate [17]; they provide high-quality protein, amino acids, and vitamins for both animal and human health [18]; and they use limited resources to produce acceptable quantities of proteins compared to conventional protein sources [13,17]. However, it is important to know that most insect products used as food components today are not much better nutritionally than traditional ones and that their nutritional content and value are actually related to the rearing substrate and the surrounding conditions [19,20].

Following Meyer-Rochow’s suggestions from as early as 1975 [21], the FAO endorses the consumption of edible insects and urges people everywhere to incorporate them into their diets [19]. By the end of the decade, it is predicted that operators of insect feed would generate more than EUR 2 billion annually [22]. We can presume that several animals have evolved to consume insects as a regular component of their diet because numerous animals, such as fish, wild birds, and free-range poultry, ingest them naturally [23]. Consequently, the use of insects as a novel, sustainable alternative feed source for livestock production is increasing. Consumer acceptance, insect-rearing methods, optimization and pretreatment of the insect breeding habitat, transfer and manufacturing, applicability in food and feed, security in the lifecycle chain, and control of the sustainability of the environment all present challenges to using insects as an alternative source of protein for animals. However, compared to other alternative proteins, insects have become feasible and affordable [24].

Eating insect products, such as ants, by poultry, is not a novel practice; it occurs naturally. It is well known that birds obtain a portion of their protein from their natural foraging behavior and eating these insect products. Menozzi et al. [25] reported that correcting some concepts and spreading proper awareness of using insect products in foods significantly increased the desire to eat such products, implying that increasing consumers’ knowledge may help to reduce fears and misconceptions about using insects as a feed source. Therefore, this review discusses the composition of insects, their nutritional value, and their role in sustaining poultry production and lowering environmental risks. It also considers some of the insects’ strengths and weaknesses, in addition to the possibility of providing insects as a major source of nutrients in the future.

## 2. Insects’ Nutritional Value

Insects are now involved in many food industries and products, such as bread, pasta, sweets (chocolates), and cosmetics [26,27,28]. Insects are a good source of protein, fat, calcium, and other important nutrients. They also have bioactive parts, such as chitin, antimicrobial peptides, and lauric acid, which help with hematological, biochemical, and immunological parameters. Numerous studies have been conducted to try and determine the nutritional benefits of insects, but because of the large number of insects (more than 2000 species) and the wide range of nutrient values, researchers attempt to determine the most significant nutritional content that has been achieved in prior studies.

### 2.1. Chemical Composition

Insects are primarily made up of protein, with fat being the second component [29]. For example, the pupal powder of the silkworm *Antheraea pernyi* contains a substantial amount of protein (71.9%) and fat (20.0%) [20,30]. With a range of 23% to 76% in protein content (on dry matter basis), the comparative amount of protein might vary significantly. Most people consider insects to be a rich source of vital amino acids. Despite the fact that the precise proportions of essential amino acids vary depending on the species and developmental phases, they generally stack up well against conventional protein sources used throughout the animal feed and high-quality sources of protein used by consumers, such as meat, dairy, and fish [16].

Triacylglycerol and phospholipids make up 80 and 20% of all fats, respectively, and fat is found in several forms [31]. An acceptable substitute for less eco-friendly fat sources might be insect fat (palm kernel). Comparable to chicken and fish, unsaturated acids have a greater impact on the fatty acid composition of edible insects than saturated acids [29]. After that, the resultant oil might be employed as an additional component [12].

Generally, insects contain a variety of polyunsaturated, saturated, and monounsaturated fatty acids, as do other animal species. The predominant saturated fatty acid is palmitic acid (C16:0), whereas stearic acid is present in varying amounts (C18:0). The predominant monounsaturated fatty acid is oleic acid (C18:1). The content of micronutrients and vitamins in insects varies between species and orders, as well as seasonally and according to the meal given [32]. The content that is accessible for each species also differs greatly [16], which could be because studies were performed at various times of the insect’s life cycle.

Insects contain enough minerals to satisfy most animals’ nutritional needs [32]. With significantly high quantities of iron and zinc, insects can provide large amounts of calcium, magnesium, manganese, phosphorus, and selenium [16]. However, insects are deficient in vitamin A, vitamin C, niacin (B3), thiamine (B1), and vitamin D. In addition, some insects contain high quantities of the B vitamins such as cobalamin (B12), riboflavin (B2), pantothenic acid (B5), and biotin [33]. Table 1 states the chemical composition of some insects [4,12,34,35,36,37,38,39].

### 2.2. Bioactive Components

#### 2.2.1. Chitin

Chitin, the second-most common polysaccharide, is a constituent of the exoskeletons of insects and is found in other species, such as crustaceans [40]. Since fish, birds, and mammals cannot synthesize chitin, it may be a priority for the immune system to recognize it [41].

Considering its biological and economic significance, chitin and its derivatives, chitosan and chito-oligosaccharides, have gained a lot of attention [42]. In previous studies addressing the activation and recruitment of innate immune cells as well as the generation of cytokines and chemokines, they have been demonstrated to enhance both the innate immune system’s and the adaptive immune system’s responses [40].

In addition, several studies have shown that chitin has additional biological advantages, such as antibacterial, antifungal, and antiviral activities, which are relevant given that it may be used as a feed additive [43]. This result can also be related to the consumption of chitin, which appears to have a bacteriostatic effect on Gram-negative bacteria such as *E. coli*, *Vibrio cholerae*, *Shigella dysenteriae*, and *Bacteroides fragilis* [44].

#### 2.2.2. Antimicrobial Peptides and Lauric Acid

Antimicrobial peptides offer a great deal of optimism in the face of the widespread problem of bacterial antibiotic resistance. Antimicrobial peptides are also thought of as growth and health promoters with moderating effects on the gut microbes that do not lead to the emergence of innate bacterial resistance. Rather than using antibiotics, antimicrobial peptide P5 can be used as a growth enhancer in broiler chicks [45]. Antimicrobial peptides produced by insects that are bactericidal may offer some defense against viruses and fungi. Flies and other insects that thrive in contaminated environments are a great source of antimicrobials [46].

Antimicrobial peptides enhance growth performance, promote food digestion and gut function, alter the intestinal microbiota, and enhance immune function in pigs and broilers with low risk of bacterial resistance [47,48]. Additionally, it is recognized that insects are a source of antimicrobial peptides with activity against both Gram-positive and Gram-negative bacteria, which may be exploited in the production of livestock [45].

Lauric acid has well-known antiviral and bactericidal properties [43]. According to Dierick et al. [49], lauric acid is particularly effective against Gram-positive bacteria, and medium-chain fatty acids have antibacterial effects on the intestinal flora.

## 3. Use of Insects in Poultry Feed

**Figure 2:**
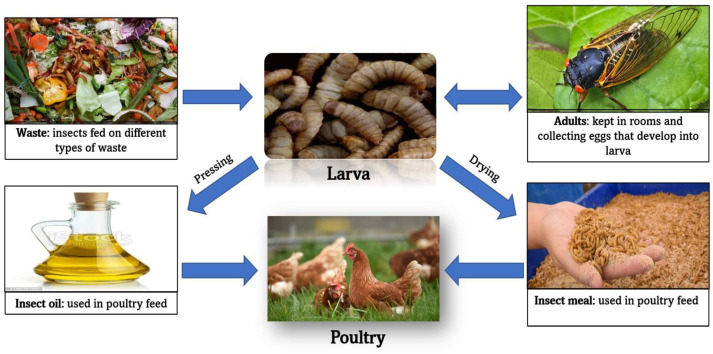
Bioconversion of waste to animal feed.

One of the most important values of insects is converting low-value content (waste) into high-value content (protein and fats). Figure 2 describes the bioconversion of waste to animal feed.

Insects are a normal and necessary component of the diet of free-ranging chickens and birds in the wild that forage for their own feed [50]. Several studies have investigated whether it would be possible to use insects as a substitute for traditional poultry feed [6]. The consumption of insects by chickens might result in a reduction in the number of antibiotics used in the poultry industry, which would be extremely beneficial considering the well-documented adverse impact that antibiotics have on humans and the environment (the development of drug-resistant species of bacteria) [16].

Due to the large number of insects used in poultry feed, and as an attempt to reach the best rates of use for them in the diets of poultry, we will try in this review to focus on the most important types of insects used globally in feeding poultry.

### 3.1. Black Soldier Fly

The black soldier fly is known scientifically as *Hermetia illucens.* Black soldier fly larvae (BSFL) could effectively transform low-value materials into higher value components including proteins, fats, and chitin, which they use to build their own bodies. In addition to this, it has been stated that they may flourish on a wide variety of substrates, some of which include low-value side streams or trash [51,52]. This is probably because the BSFL digestive tract has a wider variety of digestive enzymes than the digestive tracts of many other insects [53]. It has been shown that black soldier fly larvae may develop on a variety of organic waste products, including ketchup, coffee products’ pulp, meat, and vegetables, despite their origins in excrement from cattle, pigs, and chickens [54]. The nutrient content in diets produced from BSFL has been observed to vary between studies. Depending on the source, BSFL can have anywhere from 40.2% to 44.3% crude protein, 15.1% to 49.0% crude fat, 7.1% to 10.2% crude fiber, 11.5% to 28.3% ash, and 5278.50 kcal/kg gross energy on a dry matter basis [34,54,55]. The calcium content of BSFL ranges from 5 to 8.1%, whereas the phosphorus content ranges from 0.5% to 1.4%. In addition, the mineral composition includes copper (6.2 mg/kg), iron (0.13% to 14.1%), manganese (245 mg/kg), magnesium (0.38%), sodium (0.14%), potassium (0.70%), and zinc (109 mg/kg) [54]. Essential amino acids such as leucine, isoleucine, and valine were abundant in BSFL, as reported by [54]. Lower concentrations of lysine and methionine were discovered by De Marco et al. [55]. In terms of fatty acids, BSFL always has a composition of about 21% lauric acid, 32% oleic acid, and 16% palmitic acid, which can be much higher depending on the rearing substrate. Due to the importance of the chemical composition of this insect, many studies have been conducted on it to reach the best rates of use for it in poultry diets.

Working on the growing of quails [56], three different diets were used: a control (basal diet), 10% BFSL, and 15% BFSL diet. Across the experimental groups, the quails had the same rates of mortality, feed consumption, and conversion ratio of feed. Neither the control group nor the group given 15% BSFL in the feed had any noticeable differences in breast meat weight. Quails that were administered defatted *H. illucens* did not show any significant impact on the gained body weight [54]. When growing quails were raised in an intense condition and fed either a defatted *H. illucens* meal or a control group diet, researchers found no significant change in the daily feed consumption compared to the standard feed [54]. Additionally, the livability of quails was not affected by the inclusion of 10% dried *H. illucens* larvae, as mentioned by Woods et al. [34]. Five inclusion levels of dried BSFL were investigated: 0%, 2.5%, 5%, 7.5%, and 10% in the starting diet, and 0%, 5%, 10%, 15%, and 20% in the grower and finisher diets by [57]. The authors discovered no statistically significant change in the overall broiler performance between treatments. During the growth stage (days 11–21), however, there were substantial differences across the five treatments in terms of the mean feed consumption, body weight, and the ratio of feed conversion. The broilers fed a 10% BSFL inclusion diet had the greatest conversion feed ratio and the highest feed consumption.

To determine how the substitution of soybean oil by BSFL affects broiler performance, Benzertiha et al. [58] conducted a study. They found no statistically significant variations between the treated group and the control in terms of the rate of growth in body weight, the amount of feed consumed, and the ratio of feed converted into gain. They also revealed that the most productive broiler diet was treated with BSFL oil at 75% and 100% of the total fat content.

Better growth rates and nitrogen balance in the starter phase were observed in Ross 308 broiler hens fed a diet containing 2.6% BSFL with an excess supply of amino acids [59]. Superior feed utilization was detected by 5% inclusion of BSFL-fed Cobb broiler chickens; however, 7.5% BSFL inclusion increased the thigh weight and reduced the pH of the meat, and 10% BSFL inclusion still resulted in better growth [60].

Broiler chickens of the Ross 308 breed that were fed a diet including 20% BSFL had higher amounts of lauric acid in their feed, which led to an improvement in the overall quality of the meat [61]. In another study, feeding male Ross 708 broiler chickens a meal that included 5% BSFL led to an improvement in the cecal microbiota population as well as performance [62]. Broiler chickens fed diets that included 5% BSFL had a lower percentage of abdominal fat; feeding broilers a diet that included 10% BSFL enhanced the weight of the carcass and breast percentage; and feeding broiler chickens a diet that included 15% BSFL enhanced the live weight, abdominal fat percentage, redness of meat, meat protein percentage, and improved the breast meat fatty acids [63]. Ross 308 broiler chickens that were fed a diet that contained 5% or 10% BSFL grew more quickly compared to the control; meanwhile, a diet including 15% BSFL reduced the feed efficiency and improved crypt depth [64]. Feeding Cobb 500 broiler chickens a diet with 2% BSFL reduced their abdominal fats, while feeding them a diet with 6% BSFL improved their protein digestion and decreased the number of *Enterobacteriaceae* in their feces; feeding them a diet with 8% BSFL improved their production performance; and feeding them a diet with 10% BSFL caused a rise in their drip loss and reduced their gizzard weight [65].

Feeding Ross 308 broiler chickens a diet supplemented with BSFL (3% or 6%) caused an improvement in the breast meat percentage and feed utilization and a decrease in weight growth [45]. Increases in lymphocytes, cell proliferation, lysozyme, survival against *Salmonella gallinarum*, and reductions in bacterial count in the liver, spleen, bursa, and cecum all point to immunomodulatory effects of BSFL at the 3% dose [66]. The growth performance, hematological parameters, carcass, and quality of meat were not impacted by a diet that contained 100% BSFL as an alternative to soybean oil; however, the breast meat saturated fatty acid to polyunsaturated fatty acid ratio improved [67]. Ross 708 broiler chicks’ breast meat cholesterol levels were lowered when 50% soybean oil was substituted with BSFL fat, but 100% BSFL fat increased the total saturated fatty acids and decreased the monounsaturated fatty acids [68].

There have been few studies conducted on the effects of *H. illucens* (BSFL) on laying hens, and the results have been confusing. Marono et al. [69] reported that *H. illucens* larvae meal can be a suitable alternative protein source for laying hens without causing negative effects on birds’ health and immunity. According to Ruhnke et al. [70], the average intake of *H. illucens* larvae (15 g/hen/day) indicated a good level of acceptance; however, the feed formulation should be adjusted for the intake of the choice fed source. Choice-feeding with *H. illucens* larvae had no effect on the hen performance or egg quality after 6 and 12 weeks, respectively. In addition, Cutrignelli et al. [71] revealed that the total replacement of soybean meal with *H. illucens* larvae meal in laying hens’ diet from 24 to 45 weeks of age resulted in higher cecal production of butyric acid while the enzymatic activities of the brush border membrane were partially reduced. Furthermore, Tahamtani et al. [72] concluded that substituting conventional feed for 20% of *H. illucens* larvae could promote egg industry sustainability and be economically advantageous if *H. illucens* larvae can be bought in bulk for less than 40% of the cost of the concentrate. The growth performance of Hy-Line Brown laying hens supplied with 3% BSFL was enhanced, as well as the apparent digestibility of crude protein and crude fat, immunoglobulin A, and glutathione peroxidase [73]. The egg weight, egg mass, nitrogen, and energy metabolizability of Lohman Brown classic laying hens served diets supplemented with 15% defatted BSFL also got better [74]. A diet that included 7.5% BSFL was fed to Shaver white leghorn hens, which led to increases in the feed consumption, body weight (27 weeks), yolk color rating, and shell thickness. On the other hand, a diet that included 5% BSFL led to a rise in body weight (23 weeks) and shank-breaking strength, but it resulted in a decrease in hen-day egg production, egg weight, egg mass, and feed intake [75]. Table 2 summarizes the most important results of the influence of black soldier fly larvae on the poultry diet [34,54,56,57,58,60,61,62,73,75].

### 3.2. Mealworm

The mealworm is known scientifically as *Tenebrio molitor*. The larvae of darkling beetles resemble brown worms and are commonly known as mealworms. Mealworms thrive in warm, dark, and moist environments, such as those found under decaying logs and leaves, and they can be found everywhere in the world. *T. molitor* has the potential to convert waste material into a beneficial source of essential nutrients including proteins, lipids, and chitin, reducing the environmental burden and contamination concerns derived from organic waste accumulation. The value of this insect is an environmentally friendly technology for the dynamically recyclable processing of biowaste and the production of a reliable source of protein.

The content of the crude protein percentage in mealworm meal (MWM) ranged from 28% to 53%, and the crude fat ranged from 5% to 33% [76]. The values for crude protein percentage in MWM ranged from 46.40 to 53.80%, as found by [76,77,78,79]. The values estimated for the crude fat percentage in MWM ranged from 21.23 to 30.09%, according to [55,75,78,79]. The diet and growth level have a role in determining the chemical composition of insect-based meals [80].

When 2.5% MWM was administered to Arbor Acres broiler chick diets, enhanced weight gain (1–10 days) and decreased albumin/globulin ratio were observed, whereas 5% MWM decreased the albumin/globulin ratio and intestinal *Escherichia coli* count [79]. Ross 308 male broiler chickens fed a diet including 4% MWM during the starter period performed better in terms of body weight, average daily growth, and rate of feed conversion [81]. Due to the prebiotic action of chitin in MWM, broiler chickens fed this diet had increased pathogenic resistance and improved immunological responses [82]. Furthermore, Shaviklo et al. [82] reported that the inclusion of MWM up to 3% in broilers’ diets, for the first 24 days of their lives, may be appropriate for achieving optimum broiler meat quality and sensory attributes.

The percentages of oleic acid and linolenic acid in the eggs of Label Hubbard hybrid free-range chickens were found to be higher when MWM was used to substitute corn gluten meal in the diet at a 7.5% level [83]. Body weight (12 days), feed consumption, and rate of feed conversion (25 to 53 days) in Ross 708 male broiler chickens fed a diet that included 15% MWM as a substitute for soybean meal, corn gluten meal, and soybean oil were all enhanced by MWM; however, body weight at 25 days was enhanced by 10% MWM and body weight at 53 days increased by 5% MWM [84]. Body weight at (12 d), weight gain at (12 d), feed consumption (1 d to 12 d), thigh percentage, and abdominal fat percentage of Ross 708 female broiler chickens were all enhanced when fed a diet that included 15% full fat MWM was used as a substitute for soybean meal, corn gluten meal, and soybean oil; nevertheless, body weight at 40 d, feed consumption (12 d to 25 d), and carcass weight were all enhanced by 5% MWM inclusion [85]. The quality of chicken meat may be controlled by using a mealworm meal. A dosage of MWM sloth at 1% suppressed the red and yellow meat color, palmitic and palmitoleic acids, linoleic acid, and saturated fatty acids, and raised oleic acid and unsaturated fatty acids in the meat [86]. The breast meat from broilers supplemented with oil from the *Tenebrio molitor* had higher levels of omega-3 and omega-6 fatty acids [87]. Table 3 summarizes the most important results of the influence of mealworm meal in the poultry diet [76,79,83,84,87].

### 3.3. Housefly

The housefly is known scientifically as *Musca domestica*. The housefly is ubiquitous, appearing in every region and weather condition. Its diet consists primarily of animal excrement and other food scraps; hence, it is often seen around animal manure. Housefly meal (HFM) has a crude protein content of 40–64% and a crude fat level of 2.5–28%. HFM comprises roughly 53.3% [35], 54.36% crude protein and 16.90% crude fat [88], 55.1% crude protein and 20.7% crude fat [89], 55.6% crude protein, and 27.9% crude fat [90]. Although the amino acid profile of HFM is comparable to that of fish meal, the most limiting amino acids, lysine and methionine, are present in significantly greater concentrations.

The manner in which insects are processed might potentially have an effect on the nutritional content of the insect meal. When opposed to oven drying, sun drying results in lower crude protein levels and a higher fat content [91]. HFM can increase the growth efficiency [92,93,94,95] and quality of broiler meat [90,96] at various doses; thus, HFM is a suitable alternative to both fish and soybean meals.

When fed to Anak broiler chickens, a diet that included 20% HFM as a substitute for fish meals led to increases in body weight, feed consumption, feed conversion efficiency, and gizzard percentage. A diet that included 40% HFM, on the other hand, contributed to a rise in weight gain, dressing percentage, and abdominal fat percentage [94]. The production of Ross 308 male broiler chickens provided 4% HFM was superior; however, the inclusion of 8% HFM to the starting diet was detrimental to the growth of the birds [95]. The body weight, feed conversion efficiency, and dressing percentage of broiler chickens were all boosted when they were provided with a diet that contained 60% HFM instead of soybean meal, and feed consumption decreased [94]. Ross 308 broiler chickens provided with a diet that included 10% HFM showed improvements in weight (28 to 35 days), feed consumption (28 to 35 days), weight gain, feed conversion efficiency, European production efficiency factor, and protein efficiency factor. On the other hand, broiler chickens provided with a diet that included 50% HFM showed reductions in body weight (21 to 35 days), feed consumption (21 to 35 days), weight gain, feed conversion efficiency, and protein efficiency factor [96].

Adding 5% HFM to the diet of Cobb 500 broiler chickens during the starter stage and 4% HFM during the grower and finisher stages increased the birds’ body weight, feed consumption, feed efficiency ratio, meat flavor, meat aroma, and meat desirability, while adding 10% HFM improved the meat juiciness and flavor. Adding 20% HFM enhanced the textural characteristics and flavor [94]. The body weight, carcass percentage, breast muscle percentage, juiciness, water-holding capacity, and reduction in thawing loss and cooking loss of Ross 308 broiler chickens fed 10% HFM were all improved [96]. Further, replacing 50% of the fish meal with HFM boosted the egg production in both Isa brown and Nera black layer hens [97]. Table 4 summarizes the most important results of the influence of HFM on the poultry diet [93,94,95,96].

### 3.4. Grasshopper/Locust

The term “orthopterans” (which means “straight wings”) refers to a class of insects that includes grasshoppers, locusts, crickets, and katydids. The capacity of these species to make noise by pushing particular body sections together is one their most distinctive characteristics. Species of the Orthoptera typically have huge bodies and larger, jump-adapted hind legs.

Orthoptera meal made from crickets, grasshoppers, and locusts is a nutritious source of protein, amino acids, fatty acids, minerals, and vitamins [98,99]. Its crude protein content varied from 49% to 64%, and the crude fat content varied from 4% to 20%. According to Ghosh et al. [99], the short-horned grasshopper (*Oxya hyla hyla*) provides about 64.57% crude protein and 2.50% crude fat; the African grasshopper (*Acanthacris ruficornis*) raised in captivity provides about 50.3% crude protein and 18.2% crude fat; and the desert locust *(Schistocerca gregaria)* includes about 50.6% crude protein and 20.3% crude fat [37]. In addition, the grasshopper (*Ornithacris cavroisi*) contains 12.21% crude fat and 47.71% protein [39]. The Chinese grasshopper (*Acrida cinerea*) has a crude protein content of 65.2% and a crude fat content of 8.1% [98]. About 52.5% of the total protein and 27.1% of the crude fat are found in grasshoppers [100].

Broiler chicks from Arbor Acres that were given a diet that replaced the fish meal with 5% or 10% grasshopper meal showed better development [42]. Sanusi et al. [101] found that grasshopper meal could be used instead of fish meal in the feed of Anak 2000 broiler chickens without changing anything. Sun et al. [102] found that giving live grasshoppers at 10% to Qinjiaoma broilers increased their live weight, carcass composition, total lipids, phospholipids, and the meat’s ability to fight off oxidation. Improvements in the feed conversion efficiency and fat apparent digestibility were seen in Indigenous chicken fed a diet that included 50% wild edible grasshopper *(Ruspolia nitidual)* meal as a substitute for fish meal, and improvements in crude protein apparent digestibility were observed in chickens fed a diet that included 100% *(Ruspolia nitidual)* meal. However, diets that included increased levels (above 25%) of *(Ruspolia nitidual)* reduced the feed consumption in Indigenous chickens [37]. When Isa Brown laying hens were given a meal that substituted 25% grasshopper (*Ornithacris cavroisi*) meal for fish meal, the Haugh unit increased. A diet that included 75% grasshopper (*Ornithacris cavroisi*) meal also enhanced the color of the egg yolk [39].

### 3.5. Silkworm

Silkworm meal (SWM) contains protein, fat, amino acids, minerals, and vitamins, making it a desirable food ingredient [103]. Silkworm pupae are a by-product of the silk industry, and they have a high nutritional content [104]. About 45.87% of the weight of spun silkworm pupae is protein, whereas 50.31% of the weight of reeled silkworm pupae is protein [105]. The numbers that have been published for fat content are as follows: 20.1% for silkworm pupae meal [103], 7.94% for spun silkworm pupae, and 25.76% for reeled silkworm pupae [105]. Khan et al. [38] also revealed that silkworms contain 54% crude protein and 2.5% crude fat. Chitin is a part of the exoskeleton of silkworms. It has about 25% crude protein, but it doesn’t have any amino acids and it is difficult to digest [103].

Various amounts of SWM can be utilized in the production of poultry feed instead of fish meal or soybean meal as an alternative. With no discernible difference, SWM successfully replaced fish meal or soybean meal in the diet of broiler chickens [103]. SWM totally replaced soybean meal in the diet of white leghorn chickens without having any impact [106]. Sonali chickens fed a diet that substituted 25% SWM for soybean meal experienced weight gain and feed, increased heart percentage, and breast meat yield, and decreased breast meat protein percentage and ash percentage. Sonali chickens fed a diet that substituted 50% SWM for soybean meal experienced an increased meat pH and polyunsaturated fatty acids [107]. Additionally, diets fed to Ross 308 broiler chickens that included 75% SWM in place of soybean meal enhanced the body weight, feed consumption, gross return per bird, profit per kilogram of meat, and minimized price of a kilogram of meat; 100% SWM had the reverse effect; 25% SWM in diets lowered the feed consumption and increased the cost per kilogram of meat; and 50% SWM decreased the profit per kilogram of meat [108].

### 3.6. Termite

After being dried in the sun, termite meal has about 42.33% crude protein, and after being roasted, it has about 47.34% crude protein. It also has roughly 41% crude fat [109].

In the feed of Indigenous chickens, termites (*Macrotermes subhyalinus* and *Macrotermes bellicosus*) were replaced with fish meal in dried or fresh form without having any impact on performance [110]. It is possible to enhance poultry digestion by including termite (*Glyptotermes montanus*) derived in feed, which raises endo-D-1,4-glucanase, avicelase, D-1,4-mannanase, and D-1,4-glucosidase enzymes in the digestive system [111].

## 4. Safety and Guidelines of Using Insects as Animal Feed

When using new feed sources, their safety must be ensured to preserve the health and performance of animals, and then to preserve human health. During some previous studies, some problems affecting the use of insects as feed were noted, which we will discuss in this part. All governments, both developing and developed, need to prioritize edible insect safety laws and rules to guarantee a hazard-free or at least diminished supply of this item to consumers at every stage of its production and distribution process [112]. Consumption habits have shifted noticeably as consumers learn more about the value and rights of healthy foods, and this is unquestionably true for edible insects and their derivative products [113].

### 4.1. Safety Issues of Insects as Feed

**Figure 3:**
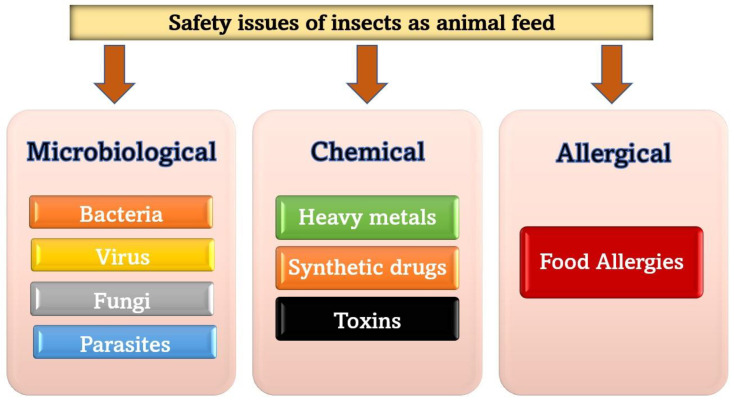
Types of safety issues face viability of insects as a source for nutrition.

Concerns have been raised about the viability of insects as a source of nutrition. Increasing the assessment of insects as a source of nutrition requires controlling for microbiological danger, chemical concerns, and potential allergies [114]. Figure 3 summarizes the safety issues that face insects as feed.

#### 4.1.1. Microbiological Safety

Insects are raised in overcrowded farms where bacteria, yeasts, and viruses can spread quickly. There has been a rise in the number of research warnings about the microbiological risks of using insects as animal feed in recent years. During a study of the microbiological status of 55 freeze-dried insects, including mealworms and locusts, researchers found that more than half of the insects contained a total amount of aerobic bacteria exceeding 6 log CFU/g, and that Enterobacteriaceae contained more than 3 log CFU/g. There are many kinds of bacteria that may be detected in insects; for example, *Clostridium* spp., *Staphylococcus* spp., and *Bacillus* (cereus group) [115].

The usage of insects as feed raises concerns about the spread of viruses. Human viruses that are closely related to those found in insects are not a threat to insect populations or human health since they cannot reproduce in insects. Some viruses, however, can be introduced with substrates into insect production units and transmitted to people. These include norovirus, hepatitis A, hepatitis E, and rotavirus [114].

Mycotoxins are fungal secondary metabolites that can cause infection in animals. They are generated by a wide variety of fungi, including *Aspergillus* spp., *Fusarium* spp., and *Penicillium* spp., However, when feeding studies were performed, no clear indication of mycotoxin buildup in insects was found. Mycotoxin buildup in insects is an area that needs more study [116].

Insects can carry and spread parasites orally, making them a possible microbiological risk. Pathogenic protozoa such as (*Giardia lamblia*, *Gongylonema pulchrum*, *Entamoeba histolytica*, *Sarcocystis* spp., and *Toxoplasma* spp.) have been shown to live in the intestines of certain insect species such as *P. americana* and *Blatella germanica* [114]. Parasite oocysts of *Eimeria tenella*, *Eimeria nieschulzi,* or *Ascaris suum* eggs were detected in the stomach of fewer than 1% of black soldier fly larvae in a study by Muller et al. [117], and thus may transmit into black soldier fly larvae feed. Although this suggests that BSFLs are mostly free of contamination, the possible danger of parasite transmission must be looked at when considering their use as animal feed [115].

#### 4.1.2. Chemical Safety

Insects are not safe from chemical pollution. Heavy metals, pesticides, and veterinary medications are all examples of chemical pollutants [110]. The presence of chemical pollutants in insects is either a result of the insect itself synthesizing a natural toxin, or of accumulation through the substrate on which they are reared. Lead, arsenic, mercury, and cadmium are just some of the heavy metals that can build up in the bodies of insects; a positive correlation between the substrate concentration of these heavy metals and the insect internal concentration has been observed [118,119]. An example of a heavy metal concentration in substrates and insects was discussed by van derFels-Klerx et al. [119]; they found that the *T. molitor* larvae cadmium concentration increased (2 mg/kg dw) as the substrate concentration increased (0.13 mg/kg).

However, they observed that for larvae provided with feed at the maximum limit of lead (0.1 mg/kg dw), the lead concentration in the larvae was below the maximum limit for lead in feed materials (<0.1 mg/kg). The concentration of heavy metals decreases during molting and metamorphosis, resulting in lower adult concentrations [119,120]. In this way, the kind of heavy metal, substrate, and insect species all influence accumulation in insects [121]. Despite the few research studies on this subject, most of them have found that residues of pesticides, veterinary medications, and hormones are broken down naturally as insects develop [114].

#### 4.1.3. Allergens

An allergy is a type of hypersensitivity reaction triggered by well-defined immunological pathways. Grasshoppers, locusts, silkworm pupae, cicadas, and bee larvae and pupae are all insects that can induce a severe allergic reaction, as reported by Van der Fels-Klerx et al. [114]. Studies on insect allergenicity are needed before they can be used extensively as animal feed. Chitin (an aminopolysaccharide), which is always present in insect cuticles, can cause allergic reactions in some people. The good news is that chitin particles that are smaller in size can mitigate the inflammatory reaction [122]. Insect consumption can cause allergic reactions in certain people because of cross-reactivity with other allergens. An allergic reaction is said to be cross-reactive if it produces symptoms in people who are allergic to unrelated proteins [123]. Earlier research has implicated proteins such as tropomyosin and arginine kinase in the cross-reactivity of allergies in edible insects [114]. Insect proteins can trigger allergies in certain people, but this may be mitigated by using innovative processing procedures.

#### 4.1.4. Antinutrients

It is also important to consider the presence of a few antinutritional substances (less than 1%) in some insect species, such as oxalates, tannins, and alkaloids, as they may affect protein digestion and mineral absorption [124].

## 5. Regulations for Insects as Animal Feed

Insects as animal feed must be governed by rules and guidelines due to several safety concerns. The insect-feeding regulatory framework varies from nation to nation because each jurisdiction has its own set of laws and historical precedents [125]. Because it is the single most important factor affecting commercial insect operations, this section is dedicated to discussing feed legislation in various nations. Unfortunately, the use of insects as either feed or food is hampered by strict prohibitions in most Western nations. There are currently insect products available on the market that are meant for human utilization and are classified as a “new food” by the European Food Safety Authority, and they must be proposed for novel food authorization in 2024. However, several countries within the EU have enacted their own laws to meet this need [126]. In North America, innovative food is subject to comparable regulations. Authoritative approvals are required in Canada from the Canadian Food Inspection Agency and Health Canada, as well as the Food and Drug Administration in the United States and the association of American feed control officers for feed ingredients definition committee [122]. The rules regarding the consumption of insects as food are summarized in Table 5 [113,122,126,127,128].

Bovine spongiform encephalopathy (BSE) is a degenerative neurological disease in cattle that can be transferred to people through the consumption of beef. This disease has a significant impact on EU legislation governing the use of insects as feed [126], because BSE probably began as a result of feeding animal proteins. In 2001, it became illegal to utilize any product derived from an animal as a feed component. After a few years, in 2013, the restriction was modified such that processed animal protein, except for ruminants, could be used in aquaculture feeding. Most farmed fish are carnivores; therefore, this shift is crucial [113]. Some animal by-products and invertebrate fats will be added to the approved list of feed ingredients in the updated catalog version. Thus, seven insect species, including house crickets (*A. domesticus*), field crickets (*G. assimilis*), banded crickets (*Gryllodes sigillatus*), yellow mealworms (*T. molitor*), lesser mealworms (*Alphitobius diaperinus*), black soldier flies (*H. illucens*), and house flies (*M. domestica*) can be utilized as ingredients in poultry, pig, and aquaculture diets.

Previous authorities have outlined the substrates that can be followed to use insect products as food. Since insect fat was allowed to be used in poultry-feeding diets, insect protein was also allowed to be used for poultry and pigs in 2021, according to IPIFF [129].

## 6. Strengths and Weaknesses

### 6.1. Strength Points

#### 6.1.1. Nutritional Composition

Many domesticated animals rely on insect prey as a part of their diet; however, the nutritional composition of insects varies widely depending on the bug kind and the substrate in which they are reared [130,131]. While insects are being researched as a potential new protein source for livestock feed, their nutritional value varies widely depending on factors such as species, rearing method, and, most importantly, substrate [132].

Insects contain a high percentage of protein (30–68%) and a somewhat complete amino acid composition [131]. Furthermore, they show promise as a fat/energy provider [84], given that they are abundant in lipids (approximately 10% to 30%), despite the fact that their fatty acid composition is extremely varied. Vitamins (notably B12) and bioavailability minerals (especially iron and zinc) are also present in significant amounts in insects [133].

#### 6.1.2. Bioactive Compounds

Insects can be used as a protein supplement to lessen the demand and dependence on traditional sources of protein, such as soybean meal and fishmeal. They also contain bioactive components, which lead to more advantageous impacts on poultry health and production [134]. Antioxidant peptides, chitin, and antimicrobial peptides found in insects have been hypothesized to enhance the immune system’s function [135], influence the animal’s gut microbiota [136], and improve animal wellbeing [137].

### 6.2. Weakness Points

#### 6.2.1. Market Price

Market pricing data are hard to come by since supply is still low, and demand is high enough to urge enterprises to negotiate individual deals depending on customer needs and volume of purchases. However, the insect-derived protein price in EU nations is higher (from 2.0 to 10.0 e/kg) [138] than outside the EU, which is largely due to the regulatory barriers and the limited industrial scale. The utilization of a wide variety of organic substrates for insect development and an increase in the manufacturing scale in the EU can lead to more affordable insect products [139]. According to the projections, the cost of insect-derived protein might drop to parity with that of fishmeal by 2023 [140].

#### 6.2.2. Polyunsaturated Fatty Acids and Minerals

As defined by the insect species and its raising environment, some species have a high polyunsaturated fatty acid content, such as grasshoppers, while many others, such as the black soldier fly, have a low content. Due to the low polyunsaturated fatty acid content in these insects, terrestrial insects may provide a problem for animal nutrition and the quality of foods generated from them [141]. Similar considerations apply to minerals, as certain species have very low calcium or phosphorus concentrations, such as locusts and grasshoppers. This trait, however, may be enhanced with proper dietary intervention in insects [142].

#### 6.2.3. Customer’s Acceptability

Acceptance does not appear to be a problem for the growth of the insect protein sector for feed, according to the limited consumer research that has been conducted so far. Nevertheless, it is important to learn if the overall acceptability, including from a sensory perspective, will be viewed better than traditional goods, and if the use of a more sustainable feed source can enhance the willingness to pay for animal products that are fed with insects. Research on whether Westerners will eat insects as food has received a lot of attention as of late, with studies focusing on factors including neophobia [143], disgust [144], familiarity (from having eaten insects before), and the difference among processed and unprocessed insects [25]. On the other hand, there was acceptance and support from others, according to Verbeke et al. [25].

## 7. Future Prospects of Insects for the Animal Feed Industry

If the insect protein business is going to realize its full potential, it must overcome three major hurdles, as stated by the International Platform of Insects for Food and Feed (IPIFF). Insect farming will first need to expand dramatically [145]. Indeed, the market price of insect meals is currently not low enough to encourage widespread adoption. Protein products derived from insects have lower production quantities by hundreds to thousands of times compared to fishmeal, high-quality soybean meal extract, and soybean meal [146]. The price of insect protein may be made more economical with other protein sources and more stable if farmers produce more of it. Specifically, stakeholders will greatly benefit from automation and control production systems since they reduce the amount of labor required to produce insects, which is an important factor [145]. Second, as was previously noted, EU livestock producers are responsible for consistently providing safe, nutritious, and high-quality goods of animal origin to EU consumers. They are also tasked with addressing social concerns, such as lowering antibiotic consumption, in order to combat the spread of antibiotic resistance. Insect farmers will need to raise the bar on product quality and nutrition to keep up with the growing demand [145]. As a last obstacle, the General Food Law must be scrupulously adhered to by insect growers. As a result of regulations, the use and consumption of insects are restricted in several EU nations [23]. Poultry and swine cannot be fed insects reared on meat, fish, or food losses from restaurants or catering companies [145], and this ban will remain in effect even if modifications are passed as planned. According to Moula and Detilleux [147], edible insects should only partially replace conventional protein sources to ensure proper bird growth and production. Accordingly, the approval of the concerned authorities can be obtained, and the use of the entomophagy industry in poultry feeding can be approved. Furthermore, public health professionals should encourage businesses to provide the opportunity to address food security issues, develop insect safety assurance systems, and work across the entire supply chain, combining experiences from other sectors in the use of insects [148].

## 8. Conclusions

**Figure 4:**
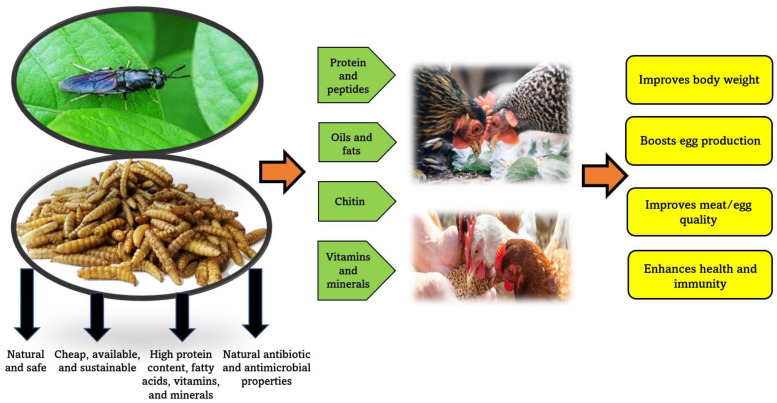
Insect products as sustainable nutraceuticals for poultry production.

The globalization of the food value chain is accelerating, and it is critical to address the challenges that come with it in a sustainable manner. Insects are currently regarded as the most promising and sustainable source of animal protein, owing to their abundance and nutritional value. Edible insect products have the potential to be sustainable food ingredients for poultry farming, based on the information presented here. These products are considered eco-friendly nutraceuticals that have several positive effects on poultry production, such as improved body weight for broilers and egg production in layers (Figure 4). Nonetheless, more research in histomorphology, histochemistry, immunohistochemistry, and molecular biology is required to ensure that these substances are effective and safe for animal and human use. More frequent cost-benefit assessments are needed to thoroughly investigate how these alternative components affect overall production costs and determine the financial effects of incorporating insects into animal diets. Additionally, consumers have become more aware and demand healthy foods that are free of synthetic ingredients. Consequently, poultry farms should prioritize animal health and welfare as well as high-quality products by using natural substances.

## Figures and Tables

**Table 1:** The average chemical composition of some insects compared to soybean meal (on dry matter basis).

Insect Name	Protein (%)	Fat (%)	Methionine (%) *	Lysine (%) *	Ca (%)	P (%)	References
Black soldier	42.3	33.5	2.1	5.7	3.2	0.9	[12,34]
House fly	52	18	2.2	6.1	0.47	1.6	[35]
Meal worm	45	30	1.5	5.4	0.27	0.78	[12,36]
Locusts	57.3	8.5	2.3	5.8	0.13	-	[37]
Silkworm	54	12	3	7	0.38	0.60	[38]
Grasshopper	47.71	12.21	-	-	-	-	[39]
Soybean meal	44	0.9	0.65	2.95	0.32	0.65	[4]

* (% to 100% protein).

**Table 2:** Effect of black soldier fly larvae supplementation (% from total feed) in poultry diets.

Bird Species	Replacements Rate (%)	Main Results	References
Growing quails	10–15%	- No differences were detected in performance and carcass traits.	[56]
Growing quails	10%	- No differences were detected in performance traits.	[34]
Growing quails	10%	- No differences were detected in performance and carcass traits.	[54]
Broilers	0, 5, 10, 15, and 20%	- 10% addition enhanced performance traits.	[57]
Broilers	75 and 100% *	- No significant difference in performance traits.	[58]
Cobb broiler	5, 7.5, and 10%	- Improved performance traits and meat quality traits.	[60]
Ross 308 broiler	20%	- Improved meat quality traits.	[61]
Ross 708 broiler	5%	- Improved performance traits and cecal microflora.	[62]
Hy-line brown laying hens	3%	- Improved performance and blood biochemistry traits.	[73]
White Leghorn laying hens	5 and 7.5%	- Improved performance and egg quality traits.	[75]

* From soybean oil.

**Table 3:** Effect of mealworm meal supplementation in poultry diets.

Bird Line	Replacements Rate (%)	Main Results	References
Arbor Acres broilers	2.5 and 5% from total feed	- Improved body weight and reduced harmful bacteria.	[79]
Ross 308 male broilers	4% from from total feed	- Improved performance traits at starter phase.	[76]
Hubbard hybrid free-range broilers	7.5% from from total feed	- Improved fat profile of meat.	[83]
Ross 708 male broilers	15% from from total feed	- Improved performance traits.	[84]
Broilers	100% from soybean oil	- Improved fat profile of breast meat.	[87]

**Table 4:** Effect of housefly meal supplementation in poultry diets.

Bird Line	Replacements Rate (%)	Main Results	References
Anak broilers	20 and 40% of fish meal	- Improved performance traits and meat quality.	[93]
Broilers	60% from soybean meal	- Improved performance and carcass traits.	[94]
Ross 308 male broilers	4% from from total feed	- Improved performance traits.	[95]
Ross 308 male broilers	10% from from total feed	- Improved growth performance, carcass, and meat quality traits.	[96]

**Table 5:** Regulation on the use of insects in some countries for feed.

Country	Authority	Regulation and Content	References
European Union (EU)	European Food Safety Authority (EFSA)	▪ Regulation: EU decisions/regulations. ▪ There must be approval for any novel feed ingredients. ▪ The EU rule allowed the use of seven insect species raised on authorized feed ingredients in aquaculture, pig, and poultry feed (*H. illucens*, *M. domestica*, *T. molitor*, *A. diaperinus*, *A. domesticus*, *G. sigillatus*, and *G. assimilis*).	[126]
United States	Federal Food and Drug Administration (FDA) and Association of American Feed Control Officials (AAFCO)	▪ Regulation: Federal Food, Drug, and Cosmetic Act (FFDCA). ▪ There must be approval for any novel feed ingredients. ▪ The use of *H. illucens* in animal feed is authorized for aquaculture as well as for all poultry.	[113]
Canada	Canadian Food Inspection Agency (CFIA)	▪ Regulation: Feeds Act and the Feeds Regulations (FAFR). ▪ There must be approval for any novel feed ingredients. ▪ The use of *H. illucens* in animal feed is authorized for aquaculture as well as for all poultry.	[126]
Republic of Korea	The Ministry of Agriculture, Food, and Rural Affairs (MAFRA)	▪ Regulation: Control of Livestock and Fish Feed Act. ▪ There must be approval for any novel feed ingredients.	[127]
China	The Ministry of Agriculture and Rural Affairs	▪ Regulation: Administrative Measures for Feed and Feed Additives. ▪ There must be approval for any novel feed ingredients.	[126]
Japan	The Ministry of Agriculture, Forestry and Fisheries	▪ Regulation: Act on Safety Assurance and Quality Improvement of Feeds. ▪ There must be approval for any novel feed ingredients.	[113]
Australia	Australian Pesticides and Veterinary Medicine Authority (APVMA)	▪ Regulation: APVMA Good Manufacturing Practice, Australian animal feed industry codes of practice, and an Australian Standard for animal feed manufacture. ▪ New feed materials do not need approval if they fulfil standards.	[122]
Nigeria	National Agency for Food and Drug Administration and Control (NAFDAC)	▪ Regulation: NAFDAC Act. ▪ There are currently no regulations regarding insect feed.	[128]
